# Optical Pump Rectification Emission: Route to Terahertz Free-Standing Surface Potential Diagnostics

**DOI:** 10.1038/s41598-017-08734-z

**Published:** 2017-08-29

**Authors:** L. Peters, J. Tunesi, A. Pasquazi, M. Peccianti

**Affiliations:** Emergent Photonics Lab (EPic), Dept. of Physics and Astronomy, University of Sussex, Brighton, BN1 9QH UK

## Abstract

We introduce a method for diagnosing the electric surface potential of a semiconductor based on THz surface generation. In our scheme, that we name Optical Pump Rectification Emission, a THz field is generated directly on the surface via surface optical rectification of an ultrashort pulse after which the DC surface potential is screened with a second optical pump pulse. As the THz generation directly relates to the surface potential arising from the surface states, we can then observe the temporal dynamics of the static surface field induced by the screening effect of the photo-carriers. Such an approach is potentially insensitive to bulk carrier dynamics and does not require special illumination geometries.

## Introduction

The diagnosis of electric surface potentials in semiconductors, arising as a result of the charged states at the surface of the material, together with the measurement of the dynamics of the surface carriers, has been at the heart of the development of many modern technologies^1^. In particular, in semiconductor technology, the map of surface carrier dynamics is of paramount importance in a number of industrial steps; for example, it is well established that the surface states strongly influence the potential of Schottky junctions^2^. Many rising technologies, such as photovoltaics and tuneable metamaterials, rely on thin-layer semiconductors and surface engineering^3, 4^, requiring fast and *in-situ* monitoring of the surface properties, capable of evaluating the dynamics of carriers and static potentials at the surface.

There are several ways (e.g. surface-tunnelling microscopy^5, 6^) to investigate the nature of surface potentials. Optical approaches have been largely investigated as effective, non-invasive alternatives, particularly suitable when ultrafast carrier dynamics need to be reconstructed. In these scenarios, the Terahertz (THz) electromagnetic radiation, lying in the electromagnetic spectrum between infrared and microwaves^7^, has been shown to be an important tool.

Optical Pump THz Probe (OPTP) is a well-established spectroscopy technique^8–11^ to monitor bulk photo-excited carrier dynamics. An ultrashort optical pulse is exploited to generate free electron-hole pairs, whereas a THz pulse is exploited to probe the excited semiconductor. The photo-carriers effectively screen the THz field, increasing the absorption of the sample: the measurement of the THz field hence provides a direct measurement of the photo-induced free electrons. By changing the relative delay between the exciting pulse and the measured THz probe, the dynamical response of the free-charges can be reconstructed. Such a technique is very popular to map the photo-carrier dynamics in bulk semiconductors, providing relevant information about their mobility and recombination time. OPTP can be used to reveal a vast array of information, such as the ultrafast carrier trapping in semiconductors^12^ and the electronic properties of nanowires^13^.

The OPTP technique can in principle be extended to measure surface photo-carrier dynamics, with an embodiment operating in reflection: a change in reflectivity of the sample can be related to the density of photo-generated electron-hole pairs, as is generally done in transmission. The optical penetration depth, when illuminating with photon energies largely exceeding the semiconductor bandgap, can be within the order of a few hundred atomic layers or lower^14^. This short depth results in the excitation of carriers in proximity of the surface. However, OPTP is in overall an approach weakly sensitive to surface dynamics. The most important limitation is that, in all practical scenarios, the THz penetration depth is usually very large, on the order of some tens of microns. This situation implies that the actual overlap between the THz decaying field and the photo-excited layer is always quite weak and very weakly dependent on the carrier dynamics in the surface field region. Most importantly, OPTP is not directly sensitive to the surface potential.

Better approaches have been proposed in literature to analyse photo-excited surface carriers. The Dynamic Terahertz Emission Microscope (DTEM) is an extension of the Laser Terahertz Emission Microscope (LTEM)^15, 16^. In these techniques a pump laser is used to excite photo-carriers. The surface current generated by such photo-carriers is the source of a THz field, which is directly generated in the photo-excited region of the sample, in striking contrast with the OPTP. The DTEM has been applied to phenomenologically describe the THz generated by surge currents in a polycrystalline silicon solar cell^17^.

Moreover, LTEM based approaches allow information on the surface potential to be extracted. Very recently, it has been demonstrated that electrically induced changes in the surface potential of materials such as gallium nitride, where the generation mechanism is dominated by surge current effects, directly affects the THz generation. Here LTEM has been used to map changes in the THz spectrum induced by the surface potential^15^. Furthermore, LTEM has since been used to probe the surface potential of electrically biased silicon^18^. Mag-Usara *et al*. proposed the “double optical pump” THz time-domain emission spectroscopy, which maps the carrier lifetimes by observing variation in the surge-current generated THz electric field when varying the delay between a THz generating pump and a second optical screening pump^19^.

A potential complexity of LTEM based approaches is that the relationship between the surface potential and the THz field is not direct: the surface field accelerates the surface photo-carriers, while the emitted THz is connected to their motional dynamics. Most importantly, in low bandgap semiconductors, THz generation mechanisms related to carrier dynamics (surge current generation and photo-Dember emission) are known to saturate quickly as the excitation increases. A recent study^20^ modelling photo-Dember carrier dynamics has attributed its saturation to the coulomb attraction. For excitation fluences of about tens of μJ/cm^2^, the THz emission is ruled by surface optical rectification.

In such regimes, the THz generation is directly related to a symmetry-breaking, static surface field which seeds optical rectification^21^. It is well established that the THz surface optical generation arises from a third order nonlinear effect involving the optical, THz and static surface field itself. The resulting THz field is then directly proportional to the surface field, with an important fallout in surface field monitoring that we will exploit in this paper. Very interestingly, in most semiconductor surfaces, such surface fields arise directly from the pinning of the Fermi Level induced by the surface states^22^. Thanks to this mechanism, the optical nonlinearity has been proposed for probing surface states in topological insulators^23^.

In this paper, we investigate the use of optical rectification to probe the surface static potential dynamics of a semiconductor by inducing a population of photo-carriers in the surface field region. An ultrashort optical pulse interacts with the surface and, thanks to the presence of the electrostatic surface field, THz radiation is emitted via a third-order nonlinearity, while a second optical ‘screening pump’ is used to generate surface photo-carriers. Differently from OPTP, where the photo-carriers screen a THz field, here the photo-carriers screen the static field directly and hence inhibits the generated THz. We will assume a screening pump with typical penetration depth on the scale of the surface field region thickness. This translates to wavelengths of 800 nm or shorter in our case of study (InAs). We name our technique Optical Pump Rectification Emission (OPRE). It is worth noting that the screening of the propagating fields has a negligible role in the OPRE.

Similar to LTEM, our approach has the benefit of directly generating the THz field on the surface of the semiconductor, with the advantage of the THz emission being directly proportional to the surface potential. Moreover, OPRE can also be efficiently applied to low-bandgap semiconductors, where the nonlinear generation is the dominating mechanism. As such, we could measure a clear linear relation between the photo-excited charges and the screening optical energy.

This approach may then open up the possibility of using THz spectroscopy for quantitative analysis of the surface potential distribution in semiconductors and, eventually, of the surface states. Moreover, our results demonstrate that it provides an efficient alternative to OPTP and LTEM for the detection of the photo-induced surface carrier dynamics.

## Optical Pump Rectification Emission

**Figure 1:**
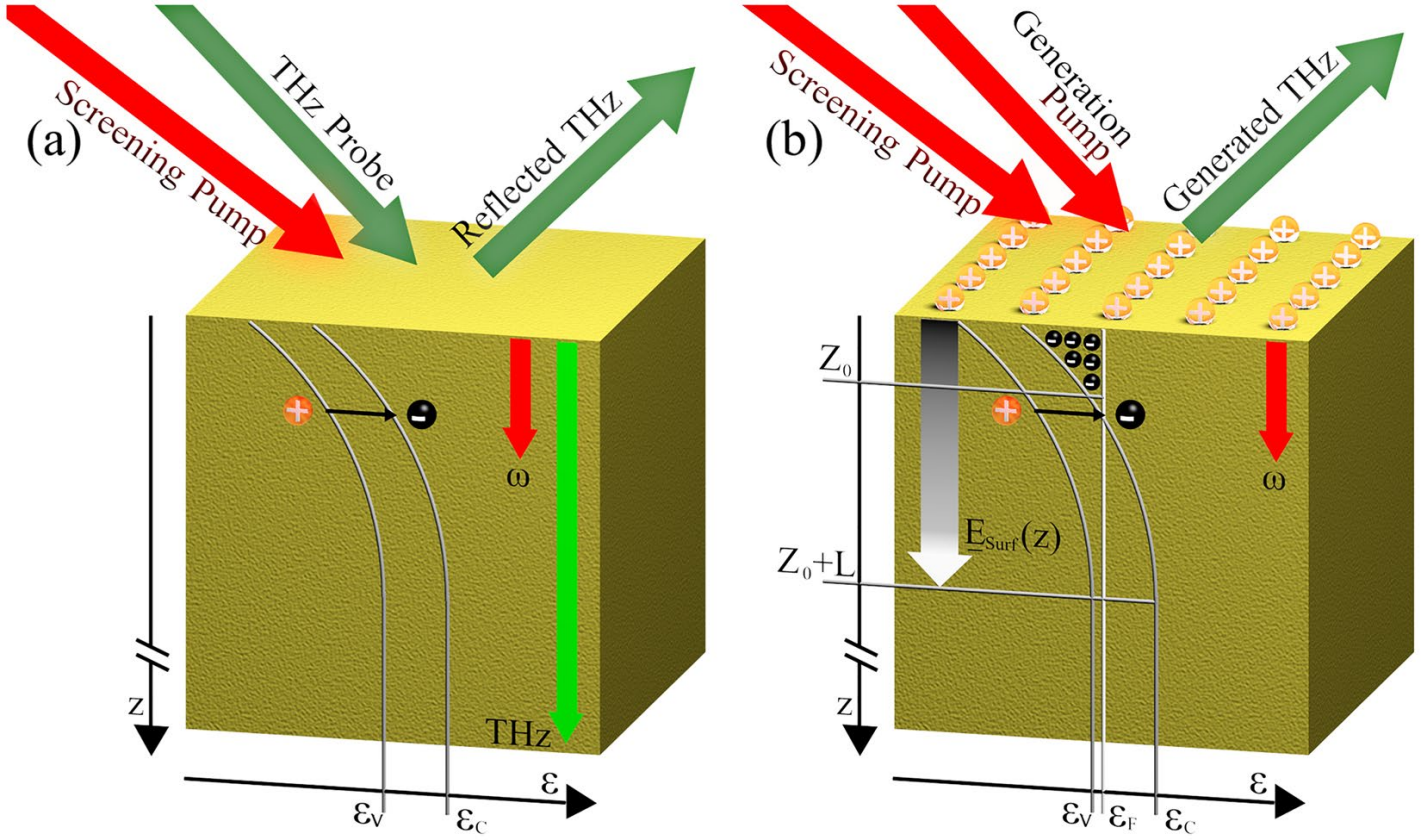
A schematic of the excitation geometry of the InAs surface, depicting the relevant physical interactions, for OPTP (**a**) and OPRE (**b**) Band diagrams along the depth direction z, with the valence and conducting energies *ε*_*v*_ and *ε*_*c*_ are indicated in both cases. (**a**) A screening pump induces free electron-hole pairs, such free-carriers are generated mostly within the skin depth of the pump, indicated with the red arrow. The concentration of free-carriers changes the reflectivity of a THz probe. The skin depth of the THz is indicated in green. (**b**) The surface field ${{\rm{E}}}_{{\rm{Z}}}^{{\rm{surf}}}$ is indicated by the gradated arrow, resulting from the balancing of the surface charges (plus signs on the top) and localised free-electrons in the surface accumulation region (0 < *z* < *Z*_0_) and the spatial charge in the depleted (*Z*_0_ < *z* < *Z*_0_ + *L*) region. Free-electrons in the conduction band are indicated by black dots with minus signs, while *ε*_*F*_ indicates the Fermi level. The THz is generated by the interaction of the generation pump with the surface field, while the screening pump also generates free-carriers in this case, effectively screening the surface field ${{\rm{E}}}_{{\rm{Z}}}^{{\rm{surf}}}$.

Before entering into the experimental details, it is useful to describe the general principle of the OPRE, together with a brief summary of the OPTP technique, which we will use in a reflection configuration as a benchmark for probing photo-induced surface carrier dynamics. Figure 1 sketches the interaction geometry in the two approaches, OPTP and OPRE in (a) and (b) respectively.

In the OPTP technique (Fig. 1(a)) a THz beam is used in reflection geometry to probe the surface carriers generated by a powerful screening pump. The pump has photon energies far exceeding the semiconductor bandgap and induces electron-hole free carrier pairs. The optical penetration depth depends on the strong single photon absorption process and, for a wavelength of 800 nm as used in this experiment is on the order of 140 nm^14^. Note that, in general, such penetration depths are not affected by the generated free-carriers until very high injections. The absorption process of the THz wave, conversely, is ruled solely by the contribution of free-carriers. Hence, the photo-induced charges screen the reflected THz wave. The absorption by free-carriers is weaker than the single photon absorption process therefore the THz penetration depth (on the order of 50 μm in undoped InAs^24^) is much larger than the penetration of the optical screening pump. We experimentally observe that this results in a small change in the reflectivity of the THz probe. In general, other important limitations arise from the fact that semiconductors usually exhibit a very high refractive index at THz frequencies^25^, hence the signal under investigation is normally superimposed with a large contribution originating from the Fresnel reflection. This aspect translates in the need of specific reflection geometries to suppress the strong linear reflection (e.g. the usage of a p-polarised field at the Brewster angle). Moreover, the optical pump can in principle produce an additional change in the THz reflection coefficient through other nonlinear mechanisms, e.g. Kerr-cross phase modulation^26^ unrelated to the surface free carrier concentration.

In the OPRE technique the THz wave is generated directly in the sample surface field region. It is important to stress that the underlying principle of surface optical rectification relies on a symmetry-breaking static (DC) surface depletion field ${\bf{E}}={{\rm{E}}}_{{\rm{z}}}^{{\rm{surf}}}\hat{{\bf{z}}}$ ^27^. The DC surface field is directed along the normal of the surface $\hat{{\boldsymbol{z}}}$, as depicted in Fig. 1(b); together with the optical generating field (**E**_**G**_(ω) in the frequency domain) it contributes to the nonlinear surface polarization **P**_**THz**_ that acts as a source for the emission of a quasi-static THz field with frequency *ω*_*THz*_ ≪ *ω*, where

1 $${{\bf{P}}}_{{\bf{T}}{\bf{H}}{\bf{z}}}={\chi }^{(3)}({{\rm{\omega }}}_{{\rm{THz}}}:-{\rm{\omega }};{\rm{\omega }},0):{{\bf{E}}}_{{\bf{G}}}(-{\rm{\omega }}):{{\bf{E}}}_{{\bf{G}}}({\rm{\omega }}):{{\rm{E}}}_{{\rm{z}}}^{surf}(0)\hat{{\bf{z}}},$$

with *χ* ^(3)^ the third order nonlinear susceptibility tensor. The surface then behaves as an effective second-order material^28^. The elements of the resulting nonlinear susceptibility tensor *χ* ^(2)^ are:

2 $${\chi }_{ijk}^{(2)}({{\rm{\omega }}}_{{\rm{THz}}}:-{\rm{\omega }};{\rm{\omega }})={{\rm{E}}}_{z}^{surf}(0){\chi }_{ijkz}^{(3)}({\omega }_{{\rm{THz}}}:-\omega ;\omega ,0).$$

The effective nonlinear coefficient is thus directly proportional to the DC surface field of the material. It is important to stress that this quadratic contribution is not related to the quadratic nonlinearity of the medium. The THz wave is then generated by an equivalent second order optical rectification of the ultrashort pulse^29^. From Eq. (1), we shall expect a trend of the THz peak field of the form ${E}_{THz}\propto {W}_{G}{{\rm{E}}}_{z}^{surf}$, where *W*_*G*_ is the energy of the THz generating pulse, i.e. the generation efficiency is:

3 $$\eta =\frac{{E}_{THz}}{{W}_{G}}\propto {{\rm{E}}}_{z}^{surf}.$$

There are two important considerations that need to be addressed. The first is that, following Eq. (1), the THz source is entirely confined within the penetration depth of the optical field (140 nm at λ = 800 nm). With the generating volume decreasing as the wavelength decreases and can be as low as ~16 nm at λ = 400 nm^14^. The second, following Eq. (3), is that the efficiency *η* directly reveals the DC surface field ${{\rm{E}}}_{z}^{surf}$.

Before describing the role of the screening pump, it is important to clarify the nature of the DC surface field, which is directly related to the electric surface states of the semiconductor. To formalise the problem and, specifically, the band bending, we consider the general case of a p-type semiconductor (i.e. with Fermi-level approaching the valence band) and a band bending enriching of a negative charge region in proximity of the surface. It is important to note, however, that in many materials (e.g. InAs, which will be used in the experiments) a significant band bending also occurs in the undoped case, due to the significant charge of the surface states.

The electron/hole surface states of a semiconductor represent a perturbation to the charge balance. The total charge *Q*_*surf*_, hosted by such surface states, needs to balance (and hence neutralise) the charge in the semiconductor. Such a charge is mostly confined in the band-bending region close to the material surface^30^, comprising the negative accumulation and Schottky depletion regions, represented in Fig. 1(b) for 0 < z < Z_0_ and *Z*_0_ < *z *< *Z*_0_ + *L* respectively.

The Schottky depletion region L is characterised by a constant density of space charge, resulting in a total charge *Q*_*Sc*_:

4 $${Q}_{Sc}=-e{N}_{A}L,$$

with *N*_*A*_ the density of ionised acceptors. Close to the surface, the bending of the bands increases and the Fermi level approaches the conduction band minimum. Here the semiconductor has a narrow accumulation region rich of free-electrons with 2-dimensional concentration *N*_*s*_^31, 32^, the total charge is:

5 $${Q}_{{Z}_{0}}=-e({N}_{s}+{N}_{A}{Z}_{0}).$$

The total charge balancing the surface charge *Q*_*surf*_ in the band-bending region is then ${Q}_{{Z}_{0}}$+ *Q*_*Sc*_. In Fig. 1(b) we reported the energy level diagram in the surface accumulation and Schottky depletion regions. The surface charge *Q*_*surf*_ is represented on the top surface of the material. The free-electron charges are sketched behind the surface. They represent a large fraction of the total charge when the Fermi level is pinned well above the conduction band minimum. The biasing potential induced by the charge of the surface states gives rise to the DC surface electric field that seeds the four wave-mixing process in Eq. (1). The DC field immediately at the surface^33^ is then

6 $${{\rm{E}}}_{z}^{surf}(z=0)=-\frac{{Q}_{{Z}_{0}}+{Q}_{Sc}}{{\epsilon }}=\frac{e}{{\epsilon }}[{N}_{A}({Z}_{0}+L)+{N}_{s}].$$

The DC surface field monotonically decreases with z in the Schottky region until it completely neutralises. In surface optical rectification, however, we can assume that a significant fraction of the generated THz can be attributed to the region between the surface and the electron accumulation layer (where the surface field is maximum and approximately constant) and is proportional to the electric field given by Eq. (6). This is also true in low-doped or intrinsic materials, as in those cases the DC surface field is only strong for z < *Z*_0_.

We can now take into account the role of the screening optical pump. When photoexcitation is included, hot carriers are induced in proximity of the surface, similarly to the case of OPTP. We will assume that, immediately after excitation, most of the photo-excited electrons are localised in the potential well and drift (because of the high mobility) towards the surface, screening the surface charges and hence the DC surface field ${{\rm{E}}}_{z}^{surf}$. By assuming that the saturation dynamics is effective within the typical THz timescales (our screening time resolution is within the order of 3 ps) the density of photo-electrons close to the surface layer can be approximated as a surface density *n*_*Ph*_, which directly modifies Eq. (6):

7 $${{\rm{E}}}_{z}^{surf}(z=0)=\frac{e}{{\epsilon }}[{N}_{A}({Z}_{0}+L)+{N}_{s}-{n}_{Ph}].$$

The carriers inhibit the DC surface field ${{\rm{E}}}_{z}^{surf}$, effectively reducing the THz generation efficiency in proximity of the accumulation region (where the nonlinear conversion is stronger because of the high field). Relevantly, at low injections, the density of screening photo-electrons is proportional to the impinging optical energy. Neglecting the very small impinging angle difference between screening pump and THz generating pump, we expect a dependence of the type:

8 $${n}_{Ph}(z)\propto ({W}_{G}+{W}_{S}),$$

where *W*_*S*_ is the energy of the screening pulse. For low energy, the modulation of the surface potential is the sole source of change, as the THz emission is not significantly related to the screening of any of the propagating fields, in stark contrast to OPTP where the photo-carriers screen the THz field directly by increasing its reflection.

Before closing this section, it is worth briefly discussing the expected dynamics of the photo-carriers. The total electromotive field for electrons is^34^

9 $${\rm{E}}(z)={{\rm{E}}}_{{\rm{z}}}^{surf}(z)+{P}_{T}\frac{dT}{dz}+\frac{e{D}_{N}}{\sigma }\frac{d{N}_{Ph}}{dz},$$

with *P*_*T*_ the thermoelectric power, *T* the temperature, *D*_*N*_ the electron diffusion coefficient, *N*_*Ph*_ the volume density of photo-generated carriers and *σ* the conductivity. At high screening injections, we expect ${{\rm{E}}}_{z}^{surf}(z)$ to be comparable or smaller than the thermoelectric and diffusion terms in Eq. (9), hence other dynamics may become relevant. It is outside the scope of this paper to discuss high-injection dynamics, but we can argue that, as the excitation increases, the depletion layer contracts because the band-bending is reduced by the accumulation of photo-electrons close to the surface. This means that we expect a saturation of the screening effect because most of the carriers will then be generated near or outside the surface field region. The motion of those carriers is then dominated by diffusion and thermal dynamics, only marginally contributing to further screening of ${{\rm{E}}}_{z}^{surf}(z)$.

Finally, the electromotive force affects both holes and electrons. In materials such as InAs, however, the mobilities of the two carriers are rather different. This creates a mostly unipolar electron flow^34, 35^. Although a unipolar flow can induce photo-Dember THz generation, such generation is quite saturated at the high excitations considered here and has negligible s-polarisation contribution^36^.

## Experimental Setup

**Figure 2:**
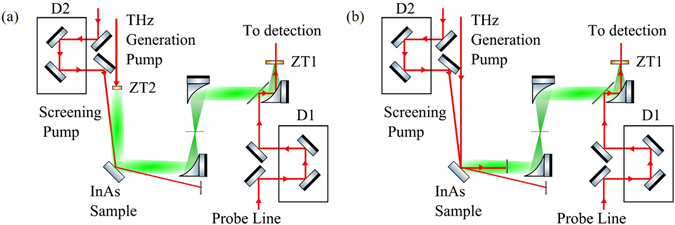
Experimental setup for OPTP (**a**) and OPRE (**b**) diagnostics. The red and green beam paths denote the 800 nm optical and THz beam paths respectively. For both setups, the THz field is measured with a standard electro-optic detection^36^, which retrieves the change of polarisation of an optical probe of energy (~1 µJ) inside a ZnTe detection crystal (ZT1) due to the THz field. A delay line D1 control the delay *t*_*d*_ between the THz and the optical probe and allows for the reconstruction of the THz waveform. (**a**) The THz is generated by a ZnTe crystal (ZT2) converting a generation pump with a pulse energy of 1 mJ. (**b**) The THz is directly generated on the sample under investigation (InAs sample) converting a generating pump with energy 0.1 mJ. In both configurations, the relative angle between the screening pump beam and the THz probe beam (**a**) or optical generating pump (**b**) is 11.9°. The p-polarised screening pump energy was 1 mJ and 0.1 mJ for (**a**) and (**b**) respectively. The delay between the screening optical pulse and the generating optical pulse *τ*_s_ is controlled with the delay line D2 in both cases.

Figure 2(a) and (b) show the experimental setup for OPTP (used as reference) and OPRE respectively. The excitation pulses are supplied by a 5 mJ-class Ti:Sa regenerative amplifier (Coherent Libra-He) generating 100 fs pulses centred at λ = 800 nm, with a 1 kHz repetition rate. The beam diameter (intensity at 1/e^2^) d = 9 mm was determined via knife-edge technique. The setup comprises of three separate beam lines, the THz excitation pump (~1.5 mJ), the screening pump (~2.5 mJ) and the optical sampling probe (~1 µJ) for the detection. A THz electro-optic detection is implemented by co-propagating the THz field and the optical sampling probe in a standard 1mm thick <110> ZnTe crystal^37^. For the OPTP, the detection crystal and the probe polarisation are set to detect the p-polarised THz field. For the OPRE a s-detection is preferred as it is in principle unaffected by any carrier-mediated generation phenomen﻿a.﻿ As in standard TDS schemes, the time-domain traces are reconstructed by changing the delay *t*_*d*_ between the THz and the optical probe. The TDS signal is measured with a balanced photo-detection unit feeding a lock-in amplifier.

The main emitter consists of a standard 1mm thick <110> ZnTe crystal for the OPTP case that generates the THz probe. The sample under investigation is an undoped 0.5 mm-thick <100> InAs crystal, which is also used for generating the THz in the OPRE case. The typical semiconductor bandgap is on the order of 0.35 eV, much lower than the photon energy of 1.55 eV used for both the generating and screening pumps, translating to a skin depth for normal illumination on the order of 140 nm^14^. This value needs to be considered as an upper boundary, and it has been found that the skin depth decreases with the impinging angle^38^ of the optical beam on the surface. The angle between the screening pump and THz probe and between the screening pump and generating pump is fixed at 11.9° for both OPTP and OPRE respectively. This results in a temporal smearing and thus a resolution of the pump delay within the order of 3 ps.

A system of delay lines allows for the independent control of the group delay in the screening pump and optical probe lines.

For the OPRE case in Fig. 2(b), the generation mechanism of the s-polarised THz emission in InAs was confirmed to be optical rectification by rotating the polarisation of the generation pump: we observed a two-fold symmetry typical of optical rectification emission from surfaces^36^. This also allowed us to exclude any relevant contribution of the photo-Dember effect, which is unaffected by a change of polarisation^36^ and was not detectable for angles of minimum generation by optical rectification. In contrast to^20^, where the sample was rotated to suppress the surface optical rectification, we oriented the surface and the generating pump polarisation to maximise the nonlinear conversion efficiency.

## Results and Discussion

**Figure 3:**
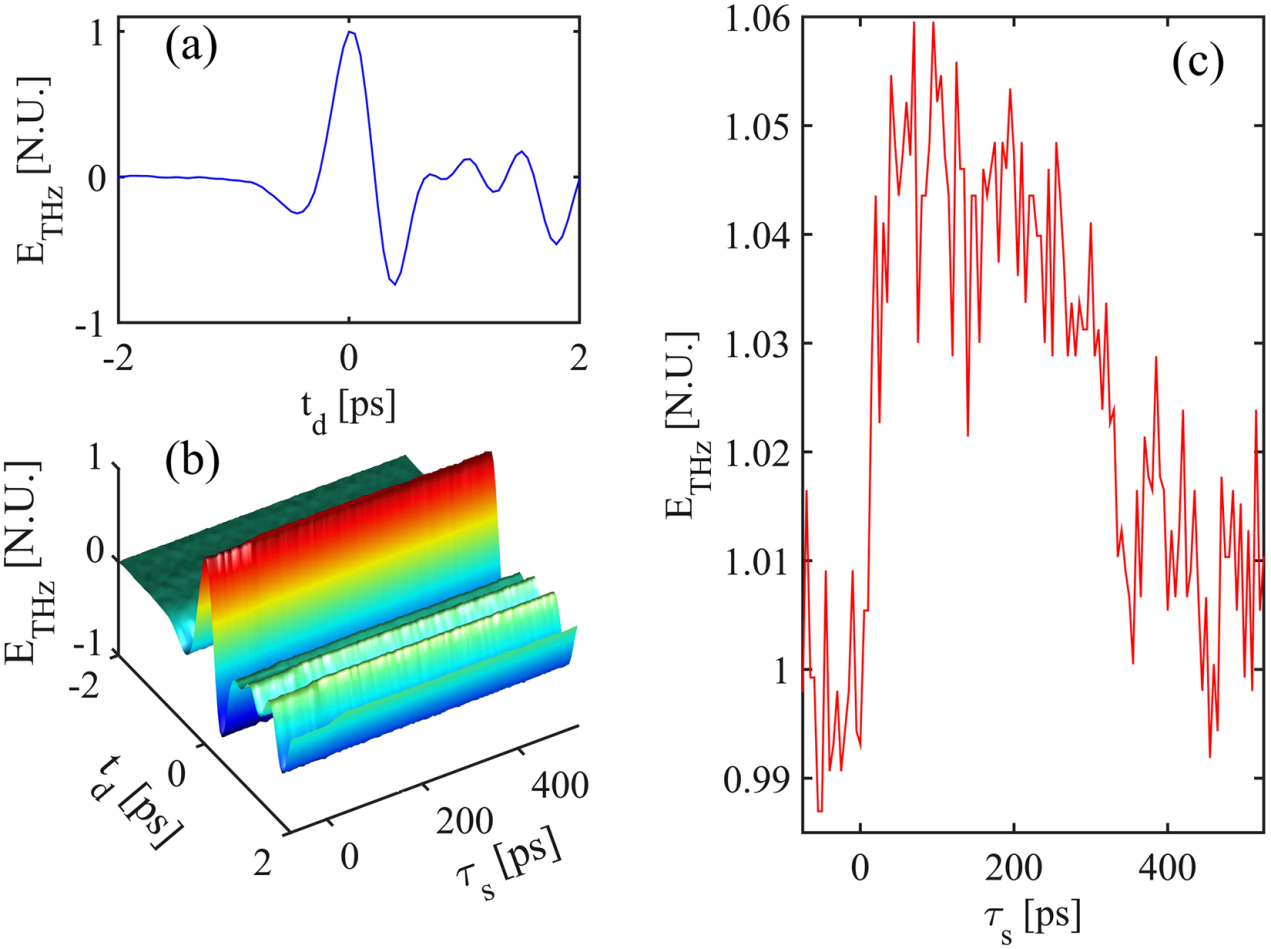
Reflective OPTP trace for an undoped <100> InAs substrate. (**a**) A typical THz waveform reflected by the InAs sample, for a generating pump energy of 1 mJ, as a function of the TDS delay *t*_*d*_. (**b**) Measured THz field as reflected from the InAs substrate as a function of the TDS delay *t*_*d*_ and screening pump delay *τ*_*s*_, for a THz pump energy and screening pump energy of 1 mJ. The delay *τ*_*s*_ = 0 represents the perfect temporal matching between the screening pump and the peak of the impinging THz wave. (**c**) Detail of the recovery of the peak THz field (*t*_*d*_ = 0) vs the screening pump delay *τ*_*s*_.

As a benchmark of our technique, we measured the photo-carrier dynamics with a reflective OPTP trace for an undoped InAs substrate. In this experiment, the THz was generated by bulk optical rectification of 1 mJ optical pulses in a ZnTe crystal. Figure 3(a) reports a typical THz wave generated in the system, reconstructed in time against the delay *t*_*d*_ between the THz pulse and the optical probe pulse in the detection scheme (Fig. 2). The reflectivity of the sample for the THz probe was increased when a screening pump of 1 mJ was overlapped in the sample. This is visible in Fig. 3(b) where we report the evolution of the THz waveform with the delay* τ*_s_ between the optical screening pump and the THz. The change of reflectivity is visible in a weak modulation of the THz wave that fades away for large delays. Reflective OPTP indeed allows for the carrier relaxation dynamics to be inferred from the change in reflectivity of the sample. The increase in reflectivity is related to the increased conductivity due to the photo-carriers generated by the screening pump.

As stated above, the interpretation of the time-resolved or frequency resolved traces is quite complex because in many practical scenarios the penetration depth of THz and optical fields are rather different. Hence, the distribution of photo-carriers along z is very inhomogeneous on the scale of the THz penetration depth. This makes the modelling of the reflection using the standard Leontovich approach difficult^39^.

Most importantly, the key drawback of this technique is that the signal variation (and therefore the change in the THz signal) is very small when compared to the total signal received, as visible in Fig. 3(b). A better visualisation of the reflectivity change is obtained by plotting the THz peak field (measured for a fixed delay *t*_*d*_ = 0), as reported in Fig. 3(c). This is the measurement with the best contrast and the relative change in THz field is approximately 5 × 10^−2^. The RMS noise of this measurement was 8.2 × 10^−3^, resulting in a signal-to-noise ratio (SNR) lower than 8.6 dB.

Widening our comparison to the reflective electro-optic sampling approach^40^ (that estimates the carrier density from the optical reflectivity) we also experimentally observed a very faint relative change of the power of the reflected THz generating optical pump, which is on the order of 4 × 10^−5^.

Summarising, the OPTP approach leads to signal contrast on the order of 5 × 10^−2^, with a low SNR of 8.6 dB, in measuring the photo-carriers effect on the reflectivity. In addition, the observed carrier dynamics show a decay on the order of ns, as visible in Fig. 3(c). Such a decay time is compatible with the carriers’ recombination time and appears mostly unrelated to the dynamics of the surface potential, which are expected to occur at lower time scales.

**Figure 4:**
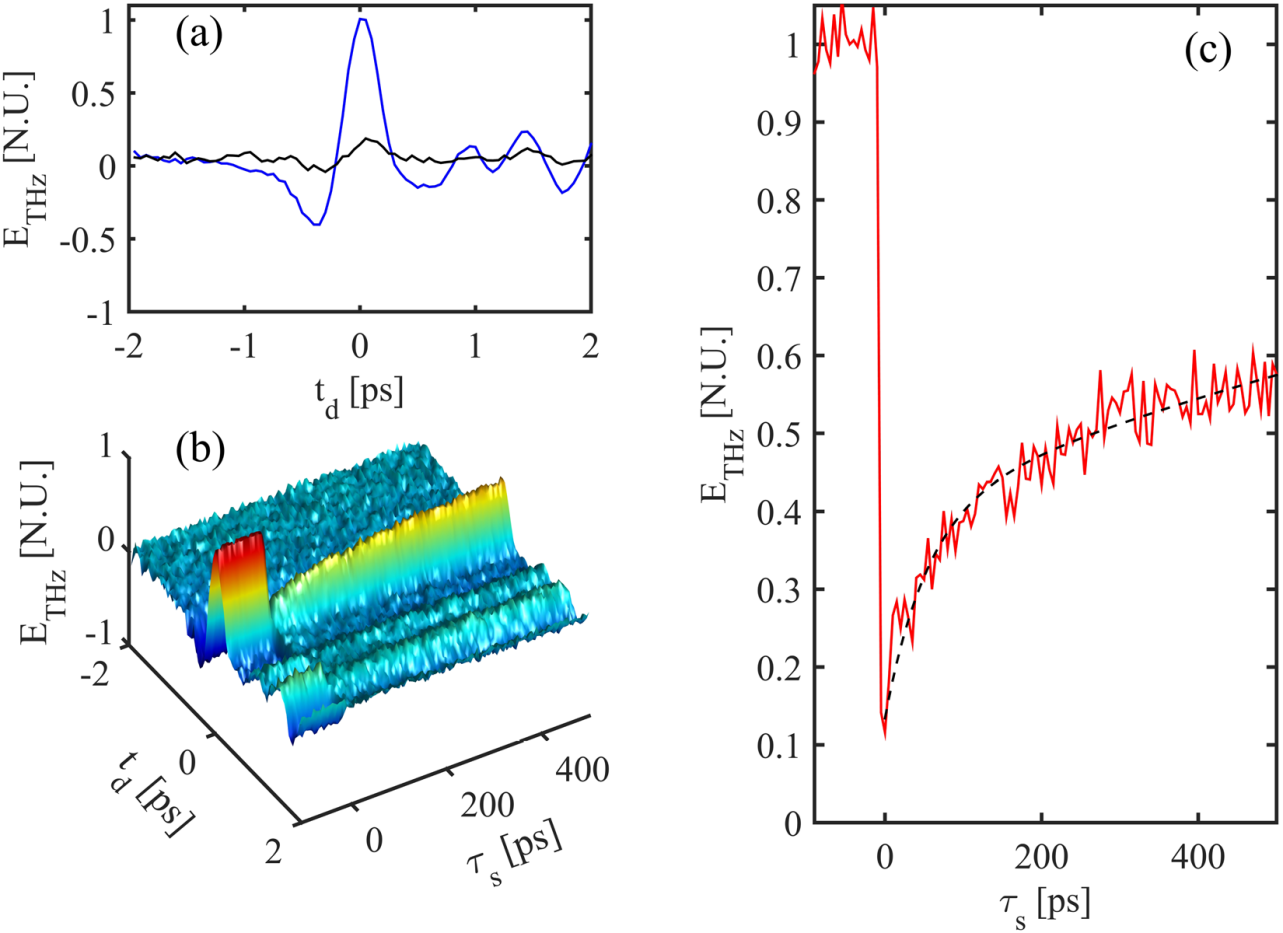
OPRE trace for an undoped <100> InAs substrate. (**a**) A typical THz waveform generated by the InAs sample, by a pump energy of 0.1 mJ, as a function of the TDS delay *t*_*d*_. The blue plot is the TDS trace for no screening pump, the black plot is for a perfectly overlapped (*τ*_*s*_ = 0) screening pump of 0.1 mJ (**b**) Measured THz field as generated from the InAs substrate as a function of the TDS delay *t*_*d*_ and screening pump delay *τ*_*s*_, for a THz pump energy and screening pump energy of 0.1 mJ. The delay *τ*_*s*_ = 0 represents the perfect temporal matching between the screening pump and the peak of the impinging THz wave. (**c**) Detail of the recovery of the peak THz field (*t*_*d*_ = 0) vs the screening pump delay *τ*_*s*_. The dashed-plot represents the best fit with a double-exponential trend. Under measuring conditions, the laser source stability introduces noise with ﻿RMS relative to the peak field estimated to be lower than 3.5%.

In the OPRE setup, the generation is obtained directly on the InAs sample with a 0.1 mJ optical pump. A typical THz trace generated with this setup is reported in Fig. 4(a), and represented by the blue plot. The THz trace for a screening pump pulse of energy 0.1 mJ at maximum overlap (*τ*_*s*_ = 0) with the generating pump is reported in black. The contrast is clearly higher than in OPTP for a much lower THz pump pulse energy (0.1 mJ) and screening pump pulse energy (0.1 mJ): at *τ*_*s*_ = 0 we observe a relative change of around 87%, with no appreciable distortion of the waveform. Under measuring conditions, the laser source stability introduces noise with RMS relative to the peak field estimated to be lower than 3.5%. This gives rise to an SNR at maximum contrast of  30 dB.

More importantly, we observe here rather different temporal dynamics. This is visible in Fig. 4(b), where we observe the change in the THz field with the delay *τ*_*s*_ for the whole temporal scale, and in Fig. 4(c), where we observe the recovery of the THz peak measured at *t*_*d*_ = 0.

Differently from the OPTP trace in Fig. 3(c), that shows a slow recovery in the ns range, the OPRE reveals a fast recovery immediately after the screening time *τ*_*s*_ = 0 We argue that OPRE perceives the modulation of the DC surface potential (which is established in a thin region), where the diffusion of photo-electrons (described in Eq. (9)) is a relevant process in InAs on the ps scale^35^, making it the source of these fast dynamics. This migration reduces the effective concentration of charges screening the DC surface electric field. A second, slower, dynamic is instead related to the recombination time, similarly to what we observed with the OPTP diagnostics. In sharp difference with the OPTP, however, the OPRE technique explicitly reveals the dynamics of carriers interacting with the DC surface field region.

For the data in Fig. 4, we found that the decaying curve can be fit by a combination of two exponential decays (reported in dashed black) with different time constants: a fast recovery time constant on the order of 50ps, that we can associate to the diffusion of the photo-carriers away from the surface-field region, and the much slower recombination time, above 1 ns (th﻿e﻿ fit of the fast recovery is quite sen﻿sitive to the estimation of the screening time). This description potentially provides another physical mechanism consistent with the need in literature to fit surface charge decay rates with two different time constants in experiments based on THz emission spectroscopy^41^, e.g. when emitted by surge currents^19^. In fact, such an experiment could face a similar decay trend as in our OPRE case.

To further analyse the capability of the OPRE experiments, we studied the effect of the screening pump energy on the THz generation efficiency *η* defined by Eq. (3).

**Figure 5:**
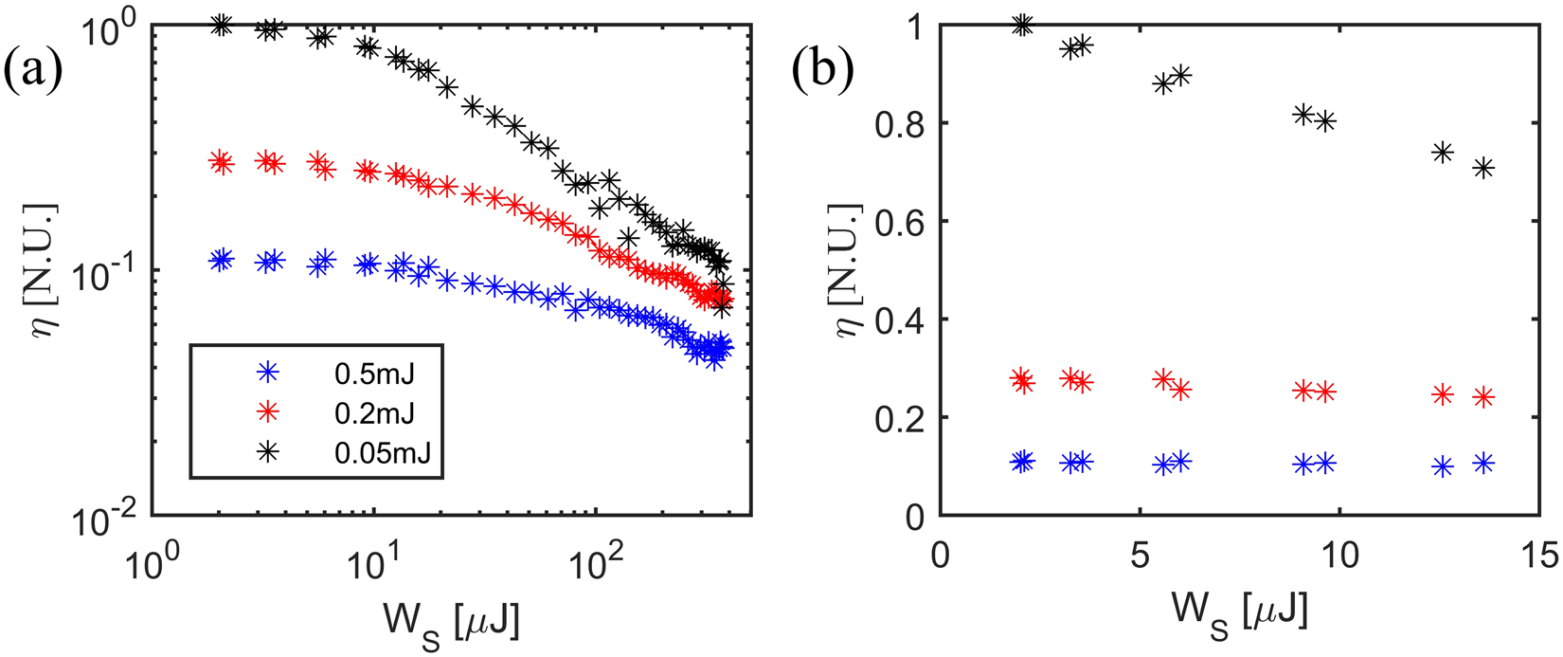
(**a**) Generation efficiency η = *E*_*THz*_/*W*_*G*_ vs. screening pump energy *W*_*s*_ for three different THz generation pump energies *W*_*G*_. (**b**) Detail of the generation efficiency at low screening energies, showing the linear trend with the screening energy *W*_*s*_.

Specifically, we considered the reduced efficiency resulting from the screening pump by measuring the THz peak field, that occurs at *τ*_*s*_ = 0 and t_d﻿_ = 0 (black plot in Fig. 4(a)). In Fig. 5 we report such a minimum for the efficiency at various screening pulse energies obtained from OPRE scans.

In line with Eqs (8, 9), as discussed previously, such a measurement is mapping the screening of the DC surface field. As visible in Fig. 5(a), the THz field initially decreases linearly with the screening pump at low energy injection (better highlighted in Fig. 5(b)) for values below 2 μJ, whereas the dependence becomes more complex at high screening energies. This result is consistent with the prediction of the linear dependence between DC surface field and screening pulse energy (i.e. a quadratic dependence between the THz pulse energy and the screening energy). Such a linear dependence also occurs with the energy of the generating pump W_G_. From Fig. 5(b), we can appreciate that, in the case where W_G_ = 0.5 mJ, the efficiency η for W_s_ ≈ 0 is approximately 35% the efficiency for W_G_ = 0.2 mJ and 10% the efficiency for W_G_ = 0.05mJ. This corroborates the hypothesis that the surface potential is screened by the surface carriers.

## Conclusions

To conclude, we have proposed a new technique for mapping the DC surface potential in semiconductors. The THz field is generated uniquely in the DC surface field-region by optical rectification and the efficiency of the process is directly dependent on the DC surface field. We tested our method by analysing the inhibiting effect in the THz generation of photo-induced free-carriers by an optical screening pump. We observed that the THz generation is strongly inhibited by the presence of the photo-carriers and that the reduction of the efficiency generation is linear with the optical screening pump energy at low injections.

In addition, when compared to the standard OPTP, our enhanced sensitivity to the surface carriers is due to the inherent physical difference between the two methods, the OPTP approach being based on a screening effect of the charges on the THz probe, that increases its reflectivity, while our OPRE is based on a modulation of the generation directly related to the screening of the DC surface field. This was also confirmed by verifying the linearity of the reduction in generation efficiency with the optical energy.

Finally, as opposed to OPTP, which is not specifically related to carrier dynamics at the surface, nor directly related to the surface potential, the OPRE is directly related to the surface potential in semiconductors. This could lead to novel methods to qualify semiconductor surfaces with potential impact in electronics, photovoltaics and opticalsensors.

### Da ta Availability

The data sets for all the experimental figures are freely accessible at https://figshare.com/articles/DataSet_Figures_3-4-5_zip/5211439.
